# Potential and limitations of applying the mean temperature approach to fossil otolith assemblages

**DOI:** 10.1007/s10641-022-01252-6

**Published:** 2022-04-12

**Authors:** Konstantina Agiadi, Rafał Nawrot, Paolo G. Albano, Efterpi Koskeridou, Martin Zuschin

**Affiliations:** 1 Department of Palaeontology, University of Vienna, Althanstrasse 14, UZA II, 1090 Vienna, Austria; 2 Department of Animal Conservation and Public Engagement, Stazione Zoologica Anton Dohrn, Villa Comunale, 80121 Naples, Italy; 3 National and Kapodistrian University of Athens, Panepistimioupolis, 15784 Athens, Greece

**Keywords:** Mean temperature of the catch, Fossil, Climate change, Baselines, Historical ecology, Conservation paleobiology

## Abstract

Evaluation of the impact of climatic changes on the composition of fish assemblages requires quantitative measures that can be compared across space and time. In this respect, the mean temperature of the catch (MTC) approach has been proven to be a very useful tool for monitoring the effect of climate change on fisheries catch. Lack of baseline data and deep-time analogues, however, prevent a more comprehensive evaluation. In this study, we explore the applicability of the mean temperature approach to fossil fish faunas by using otolith assemblage data from the eastern Mediterranean and the northern Adriatic coastal environments corresponding to the last 8000 years (Holocene) and the interval 2.58–1.80 Ma B. P. (Early Pleistocene). The calculated mean temperatures of the otolith assemblage (MTO) range from 13.5 to 17.3 °C. This case study shows that the MTO can successfully capture compositional shifts in marine fish faunas based on variations in their climatic affinity driven by regional climate differences. However, the index is sensitive to methodological choices and thus requires standardized sampling. Even though theoretical and methodological issues prevent direct comparisons between MTO and MTC values, the MTO offers a useful quantitative proxy for reconstructing spatial and temporal trends in the biogeographic affinity of fossil otolith assemblages.

## Introduction

**Fig. 1:**
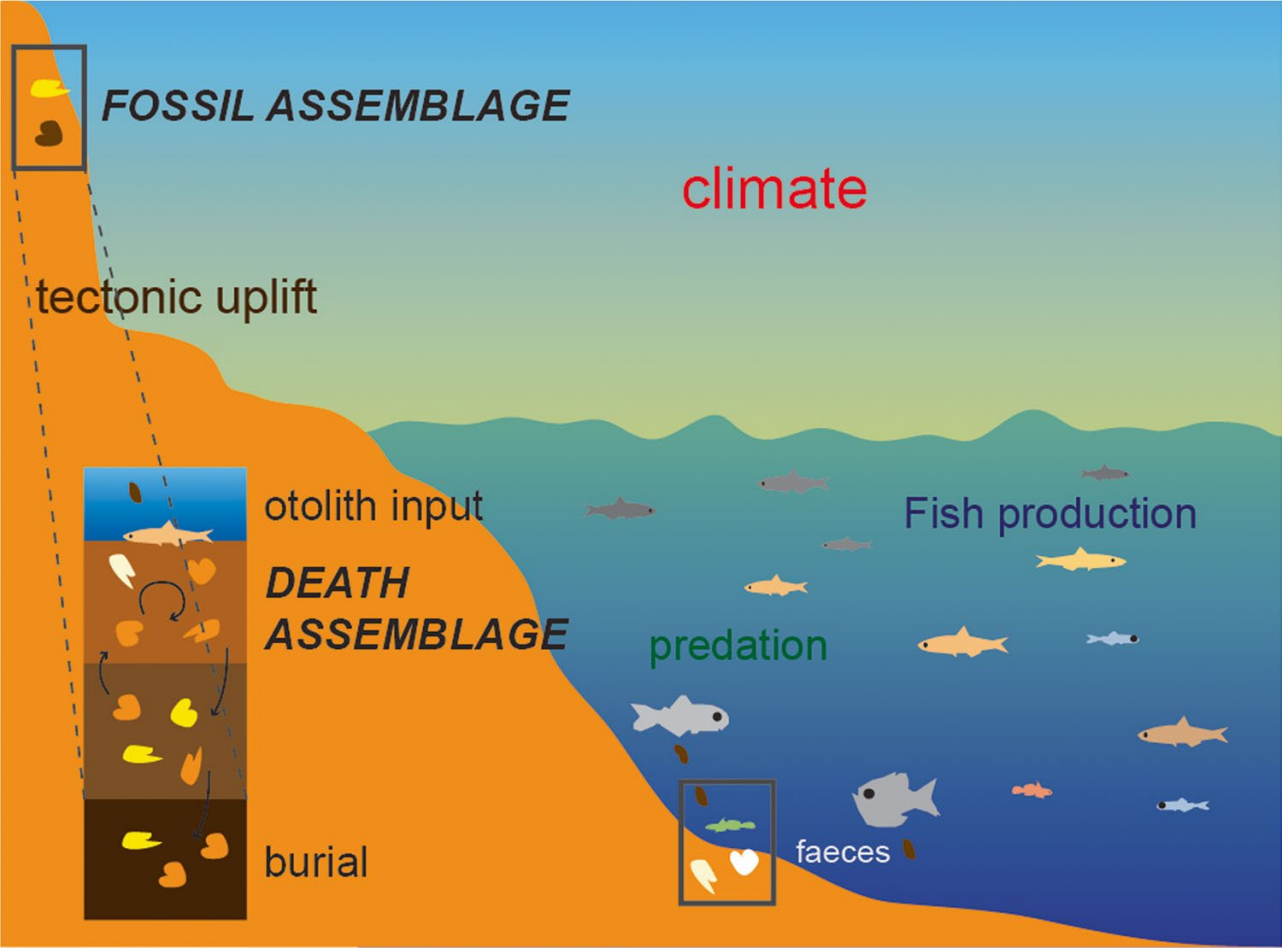
Schematic model of the pathways leading to the formation of death and fossil otolith assemblages: Otoliths are input to the seabed through the disintegrated fish skeletons or the feces of their predators, where they suffer taphonomic alteration and mixing due to the action of currents and borrowing animals. The otolith assemblages collected from the uppermost part of the seabed constitute death assemblages. After these otoliths have been buried and are no longer actively mixed — although they may still undergo chemical alteration — they finally form fossil assemblages. Tectonic uplift brings these sediments, and the fossil assemblages within them, to the surface and allows us to study them on land

Fish move to higher latitudes and deeper waters in response to climate change (Chaikin et al. 2022; Last et al. 2011; Parmesan and Yohe 2003), but the absence of baselines that precedes climate warming and other major human pressures prevents us from realizing the full magnitude of these changes. Such pre-industrial baseline datasets are often lacking in most marine ecosystems, but information can be extracted from multiple alternative sources such as historical fishers’ logbooks, museum collections, and the archeological and fossil record (Kidwell 2015; Kosnik and Kowalewski 2016). In particular, the most recent (Holocene and Pleistocene) fossil record and surface death assemblages (skeletal remains accumulating on the seabed) are proving precious for assessing the pre-human natural variability in the composition of marine faunas, and thus quantifying major shifts in their structure and functioning (e.g., Albano et al. 2021; Kowalewski et al. 2015; Steger et al. 2021; Tomašových et al. 2020; Tomašových and Kidwell 2017). Fossil assemblages, deposited, and buried within sediments (Fig. 1) are time-averaged images of past faunas, and they offer the only direct source of information on how the ecosystem operates in extreme climate states or climatic transitions. On the other hand, death assemblages of skeletal remains (such as fish bones, otoliths, scales, teeth) are recovered from the sediments currently accumulating on the surface of the seabed; Fig. 1) and are also time-averaged over decades to millennia, thereby capturing the communities from the recent past and reflecting active processes. They can, therefore, serve as baselines for comparison with the present-day fauna.

Fish sagittal otoliths (herein simply referred to as “otoliths”), the incremental and aragonitic biomineralisates in the inner ear of teleost fishes, have species-specific morphology that enables species-level identification (Nolf 1985) as well as estimation of important traits such as body size (Edelist 2014). Otolith death and fossil assemblages preserved in marine sediments enable reconstructing long-term changes of fish assemblages. Recent studies on the Holocene and Pleistocene marine fish faunas based on otolith death assemblages forming on the seabed (Agiadi and Albano 2020; Elder et al. 1996; Gaemers and Vorren 1985; Jones and Checkley 2019; Lin et al. 2019, 2018, 2017, 2016; Schwarzhans 2013) or derived from tectonically uplifted marine sediments (Agiadi et al. 2019, 2018; Aguilera and Aguilera 1999; Girone et al. 2006; Girone and Varola 2001; Schwarzhans and Ohe 2019) have shown that the required pre-industrial baseline data and past analogues of biotic responses to climate warming are within our reach.

A robust tool for easily monitoring the effect of climate warming on global fisheries is the mean temperature of the catch (MTC) index (Cheung et al. 2013). The MTC is the biomass or abundance-weighted mean temperature of the fisheries catch in a given area, based on the average preferred temperature of the exploited species that was inferred through species-distribution modeling (Cheung et al. 2013). An increase in the value of this index is considered to reflect the shift of species distributions to higher latitudes due to warming, leading to a change in the composition of the catch (Cheung et al. 2013). Although the MTC index completely relies on the availability and quality of fisheries catch data, it has been successfully applied to detect the impact of climate change on marine fisheries around the world (e.g., Dimarchopoulou et al. 2021; Fortibuoni et al. 2015; Gianelli et al. 2019; Leitão et al. 2018; Liang et al. 2018; Maharaj et al. 2018; Pauly and Liang 2020; Stergiou et al. 2016), even using archaeological data (Hillis et al. 2022).

In search of pre-industrial baseline data, we explore the applicability of the mean temperature approach to otolith death and fossil assemblages. We define here the mean temperature of otolith assemblages (MTO), in analogy to the MTC, as the mean inferred temperature preference of the species in an otolith assemblage weighted by their relative abundance. Does the MTO capture regional differences in climatic conditions that are expected to have an impact on the composition of the fish faunas? To address this question for the Mediterranean Sea, we use the otolith assemblage data derived from coastal areas in the eastern Mediterranean (samples of Holocene age from the Israeli coast covering the last ~ 8000 years, and Early Pleistocene samples from the island of Rhodes, whose age is between 2.58 and 1.8 Ma B. P.) and the northern Adriatic Sea (Holocene samples from the Gulf of Trieste, off Piran; the last ~ 7600 years). These are the only currently available quantitative abundance data for the shallow marine otolith assemblages in these regions. We compare the estimated MTO values for the three study areas, considering that: (a) the northern Adriatic and the coast of Israel represent two temperature extremes within the Mediterranean (Minnett et al. 2019), and (b) during the Early Pleistocene, the global and Mediterranean climate was colder than today, similar to pre-industrial values (Burke et al. 2018; Crippa et al. 2016) and exhibited large periodic oscillations (distinct glacial and interglacial periods) in the scale of up to 4–5 °C (Herbert et al. 2015), which we expect to be reflected in the MTO values.

## Material and methods

**Fig. 2:**
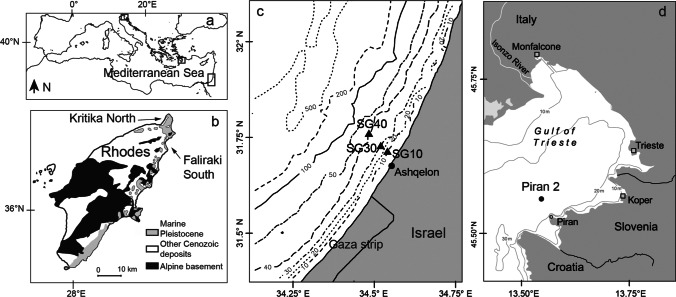
Origin of the otolith fossil and death assemblages. (a) Map of the Mediterranean area indicating the locations of Rhodes Island and the Israeli coast. (b) Simplified geological map of Rhodes Island (modified from Quillévéré et al. 2016) with the locations where the fossiliferous sediments appear and were sampled for this study. (c) Bathymetric map of the Mediterranean coast of Israel with the locations of the four stations where the sea-bottom sediment grab samples were obtained. (d) Map of the northern Adriatic indicating the sampling station location at the coast of Piran, Slovenia

The fossil and death assemblage otolith data derive from two areas in the eastern Mediterranean and one in the northern Adriatic Sea (Fig. 2a). The (paleo)environmental conditions at each locality are presented in Table 1, and the species’ abundances in the otolith assemblages are shown in Table 2.

**Table 1:** Environmental parameters in the study area at each sampled level. Pleistocene paleoenvironmental parameter estimates by Agiadi et al. (2019) and Moissette et al. (2016)

Parameters	Early Pleistocene			Holocene	Holocene		
	Faliraki South (Greece)		Kritika North (Greece)	Piran (Slovenia)	Israel shelf		
	FS2	FS3	KN6	PIR2	SG10	SG30	SG40
Setting	Upper circalittoral	Upper circalittoral	Upper circalittoral	Upper circalittoral	Infralittoral	Upper circalittoral	Upper circalittoral
Depth (m)	10–15	10–15	40–60	22.7	10	30	40
Substratum	Rock–sand	Sand	Sandy mud	Muddy sand	Sand	Sand	Sandy mud
Climate	Subtropical	Subtropical	Subtropical	Temperate	Subtropical	Subtropical	Subtropical

**Table 2:** Abundances of the species in the studied otolith death and fossil assemblages. Numbers in parentheses indicated the sieving fraction in mm

Family	Species	SG10 (> 0.5)	SG30 (> 0.5)	SG30 (> 1)	SG40 (> 0.5)	PIR 2 (> 1)	FS2 (> 0.25)	FS3 (> 0.25)	KN6 (> 0.25)
Congridae	*Ariosoma balearicum*	19	5	11	1				
Congridae	*Conger conger*		4				2	3	1
Congridae	*Panturichthys* sp.		2						
Engraulidae	*Engraulis encrasicolus*	19	135		17	1	1		
Clupeidae	*Sardina pilchardus*		2						
Clupeidae	*Sardinella aurita*	1	2	1					
Clupeidae	*Sardinella maderensis*	1	4						
Clupeidae	*Sardinella* sp.					1			
Clupeidae	Clupeidae indet		1						
Gonostomatidae	*Cyclothone braueri*		1						
Phosichthyidae	*Vinciguerria poweriae*		1						
Synodontidae	*Saurida undosquamis*		1						
Myctophidae	*Ceratoscopelus maderensis*								1
Myctophidae	*Diaphus* sp.						6	6	1
Bregmacerotidae	*Bregmaceros nectabanus*				2				
Gadidae	*Phycis blennoides*							2	
Carapidae	*Carapus acus*						1	13	1
Ophidiidae	*Ophidion barbatum*							1	11
Ophidiidae	*Ophidion rochei*		3	1					
Bythitidae	*Grammonus ater*							1	
Apogonidae	*Apogon imberbis*		1	2					
Apogonidae	*Apogon* sp.		1				4	12	
Pomacentridae	*Chromis chromis*		2			1	7	19	1
Gobiidae	*Amblygobius albimaculatus*		1		2				
Gobiidae	*Aphia minuta*	1	3						1
Gobiidae	*Callogobius* sp.		3						
Gobiidae	*Chromogobius zebratus*		2	1			10	31	
Gobiidae	Gobiidae indet. A		7		1				
Gobiidae	Gobiidae indet. B							18	4
Gobiidae	*Gobius auratus*		2	2	1				
Gobiidae	*Gobius bucchichi*					7			
Gobiidae	*Gobius cobitis*				1	10	5	1	
Gobiidae	*Gobius couchi*					4			
Gobiidae	*Gobius geniporus*							4	
Gobiidae	*Gobius niger*				1	82			
Gobiidae	*Gobius paganellus*	1	4	21	1	28		5	6
Gobiidae	*Gobius* sp.A		74	12					
Gobiidae	*Gobius* sp.B					3	26	58	20
Gobiidae	*Gobius vittatus*							1	
Gobiidae	*Deltentosteus quadrimaculatus*		1	1	2	1	3	12	69
Gobiidae	*Lesueurigobius friesii*		26	1	13			2	21
Gobiidae	*Lesueurigobius sanzi*								4
Gobiidae	*Lesueurigobius* sp.		7	1					
Gobiidae	*Lesueurigobius suerii*		6	4	12			3	
Gobiidae	*Oxyurichthys petersii*		4						
Gobiidae	*Pomatoschistus marmoratus*	2	17			2			
Gobiidae	*Thorogobius macrolepis*		6	2	2				
Gobiidae	*Zebrus zebrus*		1						
Atherinidae	*Atherina* sp.							2	
Belonidae	*Tylosurus* sp.	1							
Carangidae	*Trachurus mediterraneus*	1	1				5		13
Carangidae	*Trachurus* sp.		1						
Citharidae	*Citharus linguatula*		3		1				
Scophthalmidae	*Scophthalmus rhombus*	1	4	1					
Bothidae	*Arnoglossus laterna*	2	7		1				5
Bothidae	*Arnoglossus rueppellii*		2						
Bothidae	*Arnoglossus* sp.		1						
Soleidae	*Microchirus ocellatus*		2						
Soleidae	*Solea solea*	1							
Cynoglossidae	*Symphurus nigrescens*		10						
Callionymidae	*Callionymus filamentosus*		2						
Trachinidae	*Trachinus draco*	1	2						1
Mullidae	*Mullus barbatus*							1	
Mullidae	*Mullus surmuletus*						1		
Mullidae	*Upeneus pori*		1						
Serranidae	*Serranus* sp.					1			
Haemulidae	*Pomadasys incisus*						3		2
Cepolidae	*Cepola macrophthalma*							3	1
Scorpaenidae	*Scorpaena* sp.					1			
Triglidae	*Chelidonichthys lastoviza*					2			
Triglidae	*Chelidonichthys lucerna*		1						
Sciaenidae	*Umbrina cirrosa*					1			
Sparidae	*Boops boops*								4
Sparidae	*Dentex dentex*					1			
Sparidae	*Dentex macrophthalma*								1
Sparidae	*Dentex maroccanus*						2		
Sparidae	*Diplodus annularis*		1						
Sparidae	*Diplodus* sp.			1					
Sparidae	*Pagellus acarne*		2			3			
Sparidae	*Pagellus bogaraveo*	1	2				12	10	
Sparidae	*Pagellus erythrinus*	1	1						
Sparidae	*Pagellus* sp.	1	1						
Sparidae	*Spicara flexuosa*		5		2				
Sparidae	*Spicara maena*		8						
Sparidae	*Spicara smaris*	2	10	1		2	4		3
Sparidae	Sparidae indet		2	1			6	21	6
Perciformes indet	Perciformes indet					1			

Fossil assemblages were obtained from sediments deposited during the Gelasian age (2.58–1.80 Ma B. P., Early Pleistocene, Quaternary) that have been tectonically uplifted and form today the cliffs and beaches of the northeastern coast of the island of Rhodes (southeastern Aegean, Greece; Cornée et al. 2019). The fossil otoliths were recovered from the sediment by wet-sieving (250-μm mesh), identified and counted. Two localities were sampled: Kritika North (level KN6) and Faliraki South (levels FS2 and FS3; Fig. 2b). The depositional environment is siliciclastic, corresponding to infralittoral–upper circalittoral settings with water depths down to 60 m, various substrata, and mostly subtropical climatic conditions (Table 1; Agiadi et al. 2019; Moissette et al. 2016). However, it is not possible to obtain an improved dating and time-constraint on these deposits, because the necessary stratigraphic evidence (i.e., bioevents, datable volcanic ash layers) are lacking.

Otolith death assemblage material was obtained in 2016 by sampling the surficial sea-bottom sediments along a 10–40-m-depth gradient off the Mediterranean coast of Israel (Fig. 2c). Five replicate Van Veen grab samples were obtained at three stations (SG10, SG30, and S40). The sediment samples were wet-sieved with 0.5-mm and 1-mm mesh, and the otoliths were isolated and identified. A random selection of otoliths from benthic and pelagic species was radiocarbon dated (details available in Agiadi et al. 2021; Agiadi and Albano 2020; and Albano et al. 2020). The otoliths range in post-mortem age from ~ 8000 years B. P. to the time of sampling, with the median age of 559 years and an inter-quartile range (a common measure of time-averaging, which reflects the degree of temporal mixing of a death assemblage within a sedimentary bed) of 1097 years (Agiadi and Albano 2020). Taphonomic examination revealed no differences in the preservation of otoliths with different shapes or from fishes with different lifestyles (pelagic versus demersal fishes; Agiadi et al. 2021). Given that their median age exceeds 500 years, these Holocene death assemblages can be considered representative of the pre-industrial fish fauna in this area (Agiadi and Albano 2020).

In the northern Adriatic Sea (Gulf of Trieste), otolith death assemblages were recovered from two Van Veen grab samples taken at 22.7-m depth at station Piran 2 (PIR2; Tomašových et al. 2018) located ~ 4 km north of the coast of Piran, Slovenia (Fig. 2d). The sediment samples were sieved only at 1-mm mesh in this case. The study area was flooded about 9500 years B. P., and the present-day sea level stabilized around 2000–3000 years ago (Amorosi et al. 2008). Today, the area is oligophotic with a skeletal muddy sand substratum, but until the nineteenth century, it was part of a euphotic hard-bottom oyster and *Arca noae* banks zone close to soft-bottom vegetated habitats that dominated the central part of the Gulf of Trieste (Mautner et al. 2018; Tomašových et al. 2018). Precise ages are not available for the otoliths in the grab samples. However, otoliths sampled from a core 20–30 cm below the surface of the seabed have been radiocarbon dated and are up to ~ 7600 years old (Nawrot et al. 2022). As the sediment at the station is well-mixed by deep-borrowing animals, we consider that the age range of the otoliths captured by the grabs encompasses the last ~ 7600 years as well.

We considered only otolith assemblages with at least ~ 50 specimens in the analysis (Fig. 2). From the analyzed samples, we excluded otoliths that were too poorly preserved. We calculated MTO for the entire otolith assemblages and for the pelagic and demersal components separately. In the case of the Holocene death assemblages from the eastern Mediterranean, we calculated MTO with and without the Lessepsian species, i.e. the non-indigenous species that have entered the Mediterranean from the Red Sea since the opening of the Suez Canal in 1869. Finally, the otolith assemblages used to calculate MTO had been obtained by wet-sieving sediment samples using different mesh sizes. In order to check if this difference in sample processing had an effect on the calculated MTO, we calculated the values separately for all otoliths in a given sample and only for those > 1 mm. This procedure was only possible for the 30-m-depth station in Israel, which had been sieved with both 0.5- and 1-mm meshes and contained enough otoliths in the larger sieve fraction for a meaningful comparison.

The MTC index is calculated for exploited fishes (Cheung et al. 2013), which generally occupy the euphotic zone. However, death and fossil assemblages capture fishes living in the entire water column that have died and their otoliths were deposited together on the seabed; the composition and species abundances in these accumulations of dead remains are controlled by the species-specific natural mortality and taphonomic processes (Agiadi et al. 2021). This also means that sediments deposited at greater depths include epipelagic fishes, as well as mesopelagic and deep-water fishes, whose inferred preferred temperatures are significantly lower than those of the surface-water fishes of the same area, simply because they live deeper in the water column. Consequently, any meaningful comparison should be made between assemblages from similar water depths. Based on the water depths and the estimated paleodepths at the sampling sites (Table 1), all the samples in our case study originate from within the euphotic zone.

Our case study is based on the assumption that the fish species in our assemblages exhibited thermal niche conservatism (Ern et al. 2017; Wiens et al. 2010; Wood and McDonald 1997), i.e., they maintained the same temperature preferences as they do today. For the species of the fossil and death assemblages, we obtained the preferred temperature mean values and ranges from Aquamaps (Kaschner et al. 2016; Table 3). Previous studies for the Mediterranean using the MTC index (Keskin and Pauly 2018, 2014; Tsikliras et al. 2015; Tsikliras and Stergiou 2014) obtained the preferred temperatures from Cheung et al. (2013). However, Cheung et al. (2013) did not provide estimated values for all species found in our fossil and death assemblages. The preferred temperatures from Aquamaps differ from those modeled by Cheung et al. (2013). However, Cheung et al. (2013) demonstrated that the species distribution modeling approach used to estimate these preferred temperatures for each species does not affect the relationship between MTC and regional SST changes. For species whose preferred temperatures were unknown or where unidentifiable to species level, we used the values of the phylogenetically closely related species that is most abundant in the corresponding study area (Table 3).

**Table 3:** Ecology of the fish species comprising the otolith assemblages

Species	Ecological data from	Annual preferred temperature (°C)	Status	Lifestyle
*Ariosoma balearicum*	*Ariosoma balearicum*	26.2	Native	Demersal
*Conger conger*	*Conger conger*	8.7	Native	Demersal
*Panturichthys* sp*.*	*Ariosoma balearicum*	26.2	Native	Demersal
*Engraulis encrasicolus*	*Engraulis encrasicolus*	10.8	Native	Pelagic
*Sardina pilchardus*	*Sardina pilchardus*	10.3	Native	Pelagic
*Sardinella aurita*	*Sardinella aurita*	18.8	Native	Pelagic
*Sardinella maderensis*	*Sardinella maderensis*	21.0	Native	Pelagic
*Sardinella* sp*.*	*Sardinella maderensis*	21.0	Native	Pelagic
Clupeidae indet	*Sardinella maderensis*	21.0	Native	Pelagic
*Cyclothone braueri*	*Cyclothone braueri*	9.3	Native	Pelagic
*Vinciguerria poweriae*	*Vinciguerria poweriae*	10.6	Native	Pelagic
*Saurida undosquamis*	*Saurida undosquamis*	26.0	Non-indigenous	Pelagic
*Ceratoscopelus maderensis*	*Ceratoscopelus maderensis*	8.6	Native	Pelagic
*Diaphus* sp*.*	*Diaphus rafinesquii*	10.8	Native	Pelagic
*Bregmaceros nectabanus*	*Bregmaceros nectabanus*	23.7	Non-indigenous	Pelagic
*Phycis blennoides*	*Phycis blennoides*	9.1	Native	Pelagic
*Carapus acus*	*Carapus acus*	19.4	Native	Demersal
*Ophidion barbatum*	*Ophidion barbatum*	13.8	Native	Demersal
*Ophidion rochei*	*Ophidion rochei*	16.5	Native	Demersal
*Grammonus ater*	*Grammonus ater*	18.8	Native	Demersal
*Apogon* spp*.*	*Apogon imberbis*	16.3	Native	Pelagic
*Chromis chromis*	*Chromis chromis*	20.1	Native	Demersal
*Amblygobius albimaculatus*	*Amblygobius albimaculatus*	28.4	Non-indigenous	Demersal
*Aphia minuta*	*Aphia minuta*	10.8	Native	Pelagic
*Callogobius* sp*.*	*Callogobius bifasciatus*	27.4	Non-indigenous	Demersal
*Chromogobius zebratus*	*Deltentosteus quadrimaculatus*	15.2	Native	Demersal
*Deltentosteus quadrimaculatus*	*Deltentosteus quadrimaculatus*	15.2	Native	Demersal
Gobiidae indet. A	*Gobius paganellus*	17.7	Native	Demersal
Gobiidae indet. B	*Gobius niger*	10.8	Native	Demersal
*Gobius auratus*	*Gobius auratus*	18.1	Native	Demersal
*Gobius bucchichi*	*Gobius bucchichi*	18.5	Native	Demersal
*Gobius cobitis*	*Gobius cobitis*	18.0	Native	Demersal
*Gobius couchi*	*Gobius couchi*	12.1	Native	Demersal
*Gobius geniporus*	*Gobius geniporus*	19.1	Native	Demersal
*Gobius niger*	*Gobius niger*	10.8	Native	Demersal
*Gobius paganellus*	*Gobius paganellus*	17.7	Native	Demersal
*Gobius vittatus*	*Gobius vittatus*	18.4	Native	Demersal
*Lesueurigobius friesii*	*Lesueurigobius friesii*	10.4	Native	Demersal
*Lesueurigobius sanzi*	*Lesueurigobius sanzi*	17.1	Native	Demersal
*Lesueurigobius* sp*.*	*Lesueurigobius friesii*	10.4	Native	Demersal
*Lesueurigobius suerii*	*Lesueurigobius suerii*	14.7	Native	Demersal
*Oxyurichthys petersii*	*Oxyurichthys papuensis*	27.9	Non-indigenous	Demersal
*Pomatoschistus marmoratus*	*Pomatoschistus marmoratus*	15.1	Native	Demersal
*Thorogobius macrolepis*	*Gobius niger*	10.8	Native	Demersal
*Zebrus zebrus*	*Zebrus zebrus*	19.3	Native	Demersal
*Atherina* sp*.*	*Atherina boyeri*	18.3	Native	Pelagic
*Tylosurus* sp.	*Tylosurus acus*	26.5	Native	Demersal
*Trachurus* spp.	*Trachurus mediterraneus*	17.4	Native	Pelagic
*Citharus linguatula*	*Citharus linguatula*	15.2	Native	Demersal
*Scophthalmus rhombus*	*Scophthalmus rhombus*	11.2	Native	Demersal
*Arnoglossus laterna*	*Arnoglossus laterna*	10.8	Native	Demersal
*Arnoglossus rueppellii*	*Arnoglossus rueppellii*	14.2	Native	Demersal
*Arnoglossus* sp*.*	*Arnoglossus laterna*	10.8	Native	Demersal
*Microchirus ocellatus*	*Microchirus ocellatus*	17.1	Native	Demersal
*Solea solea*	*Solea solea*	11.4	Native	Demersal
*Symphurus nigrescens*	*Symphurus nigrescens*	13.9	Native	Demersal
*Callionymus filamentosus*	*Callionymus filamentosus*	24.0	Non-indigenous	Demersal
*Trachinus draco*	*Trachinus draco*	10.9	Native	Demersal
*Mullus barbatus*	*Mullus barbatus*	14.2	Native	Demersal
*Mullus surmuletus*	*Mullus surmuletus*	10.2	Native	Demersal
*Upeneus pori*	*Upeneus pori*	25.6	Non-indigenous	Demersal
*Serranus* sp*.*	*Serranus cabrilla*	14.4	Native	Demersal
*Pomadasys incisus*	*Pomadasys incisus*	23.1	Native	Pelagic
*Cepola macrophthalma*	*Cepola macrophthalma*	11.9	Native	Demersal
*Scorpaena* sp*.*	*Scorpaena notata*	14.6	Native	Demersal
*Chelidonichthys lastoviza*	*Chelidonichthys lastoviza*	11.8	Native	Demersal
*Chelidonichthys lucerna*	*Chelidonichthys lucerna*	9.8	Native	Demersal
*Umbrina cirrosa*	*Umbrina cirrosa*	18.0	Native	Pelagic
*Boops boops*	*Boops boops*	17.8	Native	Pelagic
*Dentex dentex*	*Dentex dentex*	18.1	Native	Pelagic
*Dentex macrophthalma*	*Dentex macrophthalma*	14.9	Native	Pelagic
*Dentex maroccanus*	*Dentex maroccanus*	13.5	Native	Pelagic
*Diplodus* spp*.*	*Diplodus annularis*	18.3	Native	Pelagic
*Pagellus acarne*	*Pagellus acarne*	14.8	Native	Pelagic
*Pagellus bogaraveo*	*Pagellus bogaraveo*	11.2	Native	Pelagic
*Pagellus erythrinus*	*Pagellus erythrinus*	17.2	Native	Pelagic
*Pagellus* sp*.*	*Pagellus bogaraveo*	11.2	Native	Pelagic
Perciformes indet	*Spicara smaris*	14.4	Native	Pelagic
*Spicara flexuosa*	*Spicara flexuosa*	14.5	Native	Pelagic
*Spicara maena*	*Spicara maena*	14.5	Native	Pelagic
*Spicara smaris*	*Spicara smaris*	14.4	Native	Pelagic
Sparidae indet	*Spicara smaris*	14.4	Native	Pelagic

In analogy to MTC, MTO was calculated as:

$${\mathrm{MTO}}_{a}=\frac{{\sum }_{i}^{n}{T}_{i}{Ar}_{i,a}}{{\sum }_{i}^{n}{Ar}_{i,a}}$$

where *a* is the assemblage, *T*_i_ is the preferred temperature of species *i*, *Ar*_i,s_ is the relative abundance of species *i* in assemblage *a*, and *n* is the total number of species in assemblage *a*. We estimated 95% bootstrap confidence intervals around the MTO values using the percentile method by resampling each assemblage with replacement 10,000 times and calculating MTO values for each iteration.

## Results

**Fig. 3:**
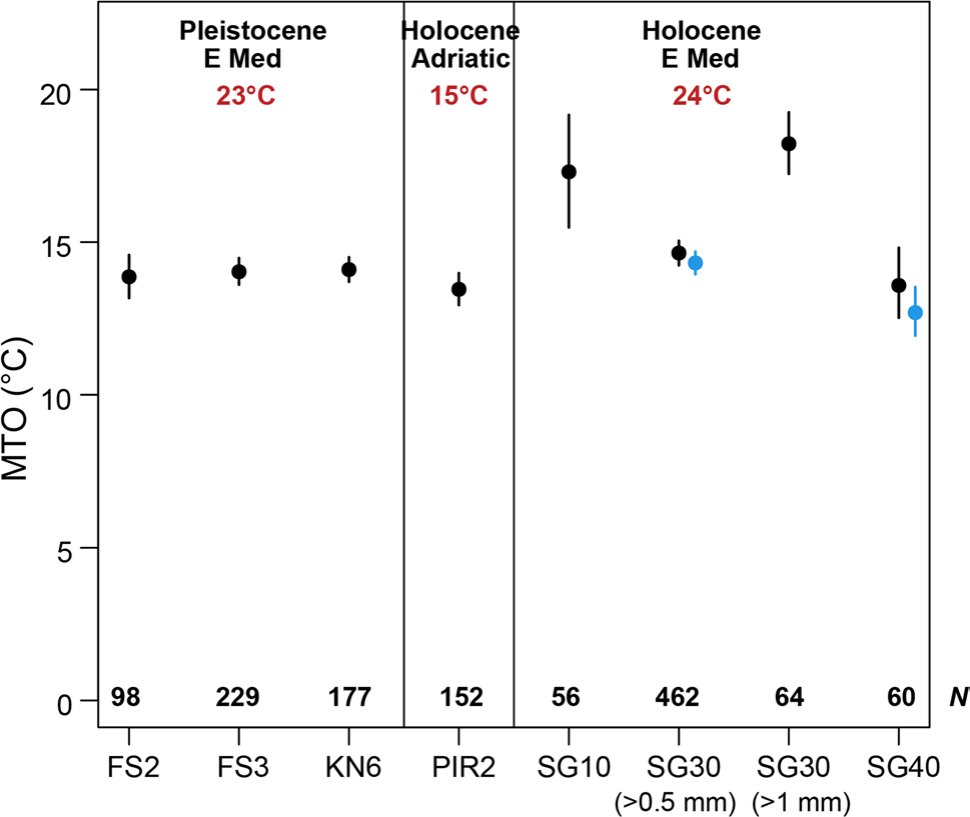
Mean temperature of the otolith assemblages from the Holocene (the last ~ 8000 years) of the eastern Mediterranean (SG10, SG30, and SG40), the Holocene (the last ~ 7600 years) of the northern Adriatic Sea (PIR2), and the Early Pleistocene (2.58–1.8 Ma B. P.) of the eastern Mediterranean (FS2, FS3, and KN6). For SG30, we also calculated the MTO for only the assemblage of otoliths > 1 mm. At 30- and 40-m depths of the Israeli coast (SG30 and SG40), where few non-indigenous species appear in the otolith assemblages, we also calculated the MTO for the assemblages without them (marked in blue). Bars indicate 95% bootstrapped confidence intervals. *N*: number of otolith specimens in the assemblage. Temperature estimates are sea surface temperature averages based on alkenone paleothermometry for each region and time interval (Adriatic Sea in the Holocene: (Giunta et al. 2001; Oldfield et al. 2003); Levantine Sea in the Holocene: (Avnaim-Katav et al. 2019; Castañeda et al. 2010; Essallami et al. 2007); Eastern Mediterranean in the Early Pleistocene (Athanasiou et al. 2017; Burke et al. 2018; Crippa et al. 2016; Lourens et al. 1992)

The Holocene MTO values for the eastern Mediterranean (Israeli coast) range from 13.6 °C (at 40-m depth) to 17.3 °C (at 10-m depth), which are slightly higher than that from the northern Adriatic (Slovenia coast) of 13.5 °C for the 22.7-m depth (Table 4; Fig. 3). The Early Pleistocene MTO at FS2 and FS3 (13.8 and 14.0 °C, respectively), which correspond to 10–15-m depth, are lower than the Holocene eastern Mediterranean values at similar depths (17.3 °C at SG10). However, the fossil assemblage KN6 corresponding to the deepest level (40–60-m depth) gives an elevated MTO (14.1 °C) compared to both the shallower Pleistocene samples and the Holocene assemblage from the comparable water depth (SG40; 13.6 °C).

**Table 4:** Mean temperature of the otolith death and fossil assemblages

Sample	Number of specimens	MTO (°C)	Weighted standard deviation (°C)
FS2	98	13.8	3.5
FS3	229	14.0	3.3
KN6	177	14.1	2.7
PIR2	152	13.5	3.3
SG10	56	17.3	7.1
SG30	462	14.6	4.4
SG30 native	450	14.3	4.0
SG40	60	13.6	4.5
SG40 native	56	12.7	3.0

Removing the non-indigenous species from the SG30 and SG40 assemblages from Israel decreases the MTO (Table 4; blue in Fig. 3). However, the difference is very small and the MTO values based on native species alone are within 95% confidence intervals for the entire assemblage, suggesting that this reduction is not statistically significant. The MTO for SG30 is primarily driven by the high abundance of the native *Engraulis encrasicolus*, which together with the rarity of non-indigenous species may explain why exclusion of the latter has only a very limited effect on the MTO values. Although *E. encrasicolus* is the most abundant species also at SG40 (Table 2), the relative contribution of the tropical non-indigenous species (*Amblygobius albimaculatus* and *Bregmaceros nectabanus*) is greater at that site, and therefore their exclusion has a slightly higher impact on the MTO value.

Isolating the coarser component (> 1 mm) of the SG30 assemblage makes it directly comparable to PIR2, which was only sieved under 1-mm mesh. Otoliths from the 1-mm fraction of SG30 give a significantly higher MTO value compared to the entire assemblage (all specimens > 0.5 mm; Fig. 3). This pattern is clearly driven by the exclusion of the dominant *E. encrasicolus* (Table 2), which only appears in the fine fraction, and whose preferred temperature (10.8 °C) is well below the average values for the assemblages. Comparing the > 1 mm component of SG30 with PIR2 drastically increases the difference between the MTO values for the eastern Mediterranean and the northern Adriatic otolith death assemblages making them more consistent with the sea surface temperature gradient between the two areas (Fig. 3).

**Fig. 4:**
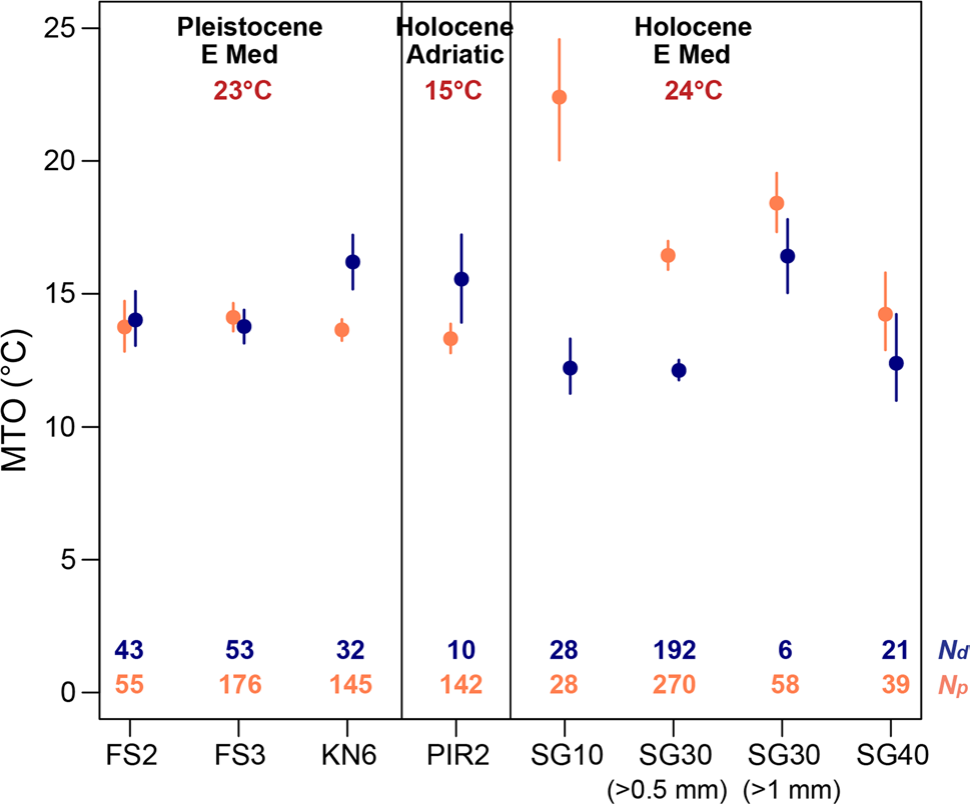
Mean temperature of the pelagic and demersal fish otolith assemblages from the Holocene (the last ~ 8000 years) of the eastern Mediterranean (SG10, SG30, and SG40), the Holocene (the last ~ 7600 years) of the northern Adriatic Sea (PIR2), and the Early Pleistocene (2.58–1.8 Ma B. P.) of the eastern Mediterranean (FS2, FS3, and KN6). For SG30, we also calculated the MTO for only the assemblage of otoliths > 1 mm. Bars indicate 95% bootstrapped confidence intervals. *N*_*p*_ is the number of pelagic fish otoliths and *N*_*d*_ the number of demersal fish otolith specimens in the assemblage. Sea surface temperature estimates as in Fig. 3

For the death assemblages from the Israeli shelf, the demersal fish MTO, particularly at SG10 (22.4 °C), is much higher than those derived from the pelagic component (12.2 °C at SG10; Fig. 4). This difference is driven by the high abundance of the demersal *Ariosoma balearicum* (Table 2) with a preferred temperature of 26.2 °C, which in fact dominates at SG10. On the other hand, in the very shallow fossil assemblages (FS2 and FS3 at paleodepths of 10–15 m), the MTO values from demersal and pelagic species are similar, whereas in the deepest assemblage (KN6 at paleodepth of 40–60 m), the pelagic species give higher MTO values (Fig. 4), as expected for a site where the difference between surface and bottom water temperature is greater. The same difference between demersal and pelagic MTO is observed in PIR2 as well (Fig. 4), even though this station represents a much shallower water depth. However, the pelagic component at PIR2 comprises only 10 otoliths, many of which could not be identified to species level, so this pattern may be partly driven by sampling bias (Table 2).

## Discussion

Our results demonstrate that the MTO can successfully capture regional variations in the composition of marine fish faunas related to climate differences, but they also point to several requirements and limitations of this approach. The MTO values calculated here for the Holocene and Pleistocene Mediterranean coastal fish assemblages range from 13.5 to 17.3 °C. As expected, based on the (paleo)climatic framework of the region (Castañeda et al. 2010; Crippa et al. 2016; Essallami et al. 2007; Giunta et al. 2001; Minnett et al. 2019), the highest MTO value corresponds to the shallowest (10-m depth) and southeastern-most site along the Mediterranean coast of Israel, whereas the lowest MTO value is recorded in the colder northern Adriatic Sea. With regard to the fossil otolith assemblages, although it is not possible to temporally constrain the Early Pleistocene samples further and directly link the otolith assemblages to specific climatic phases, their MTO range is small (13.8–14.1 °C; Table 4), especially considering the average sea surface temperature in the eastern Mediterranean during this interval (23 °C; Athanasiou et al. 2017; Burke et al. 2018; Crippa et al. 2016; Lourens et al. 1992). It is possible therefore that the observed Early Pleistocene MTO values correspond to the same type of climatic regime, specifically glacial periods. Alternatively, and despite the reconstructed shallow paleodepths of the seabed in the study area (Agiadi et al. 2019), the assemblages include species from deeper environments occasionally visiting the area, specifically mesopelagic myctophids (Table 2), which decreases the MTO.

Furthermore, the MTO is increased by the inclusion of Lessepsian species, as expected since these species are tropical and have very high preferred temperatures (Table 3). The eastern Mediterranean marine fauna is rapidly becoming more tropical due to climate change and the opening of the Suez Canal, which allows warm-water species from the Red Sea to enter into and establish populations in the basin (Edelist et al. 2013), while shifting the distribution of species preferring cooler temperatures to northern latitudes (Givan et al. 2018). In the Mediterranean, MTC based on landings has been found to increase between 1970 and 2010 by 0.56, 1.05, and 0.29 °C per decade in the western, central, and eastern sub-basins, respectively (Tsikliras and Stergiou 2014), by 0.25 °C per decade in the northeast Aegean (Keskin and Pauly 2014), by 1.01 °C, and 1.17 °C per decade for the Aegean and Ionian Seas, respectively (Tsikliras et al. 2015), and by 0.48 °C per decade in the northern part of the Levantine Sea (Keskin and Pauly 2018). These estimates were made without considering the non-indigenous tropical Lessepsian species, although they now constitute a significant proportion of the fish population, especially in the eastern Mediterranean (CIESM 2021; Edelist et al. 2013, 2011) and their inclusion in the calculation would probably have increased the estimated MTC. Although our results show a decrease in MTO when non-indigenous species are excluded from the calculation for the Holocene death assemblages, this decrease is very small due to rarity of Lessepsian species in these assemblages compared to native taxa. Specifically, dominant species, such as *E. encrasicolus* in the Israeli death assemblages at 30- and 40-m depths, drive the MTO values.

Our results provide also some practical guidelines for future studies relying on the MTO approach. In particular, we have shown that isolating size fractions in individual samples gives drastically different MTO values (Fig. 4). Similarly, mesh size has been shown to affect the estimation of a variety of ecological parameters for both fossil (e.g., Bush et al. 2007) and living assemblages, including surveys of fish communities (e.g., Bethke et al. 1999; Godø and Walsh 1992; Millar and Walsh 1992).This means that comparing death and fossil otolith assemblages based on MTO cannot be done without standardizing sample processing, specifically sieve size.

Additionally, the bathymetry of the study area influences the MTO values. This becomes obvious by the differences in MTO of pelagic versus demersal fishes, especially in the deepest assemblages (Table 4; Fig. 4). Pelagic assemblages give MTO values closer to the mean annual sea surface temperatures in the Holocene assemblages, particularly in the shallower sampling stations (Fig. 4). However, MTO based on pelagic species shows a decreasing trend along the Israeli depth gradient: the MTO at 10-m depth is 17.3 °C, but only 13.6 °C at 40-m depth (Table 4), while the sea surface temperature for this area is estimated at 24 °C on average (Avnaim-Katav et al. 2019; Castañeda et al. 2010; Essallami et al. 2007). We hypothesize that this trend is caused by the inclusion of pelagic species living in deeper levels of the water column in the assemblages from the deeper stations.

A potential limitation of MTO comes from a basic paleoecological assumption of niche conservatism. Fish show thermal plasticity when faced with gradual seawater temperature change (Crozier and Hutchings 2014; Loisel et al. 2019; Ryu et al. 2020), and therefore may be expected to have shown somewhat different temperature preferences between long-term climate perturbations over geological timescales, such as the glacial and interglacial stages. Although the assumption that fish species maintained the same traits in the geological past as today is a good starting point in paleoecological research (Eduardo et al. 2018; El-Sayed et al. 2021), it may not hold true, and this should be considered when using MTO. Nevertheless, MTO, as MTC, accepts that fish species’ first respond to climate change by shifting their distribution, rather than change their temperature preferences, and indeed this seemed to be the case as well during the Pleistocene climatic perturbation in the eastern Mediterranean (Agiadi et al. 2018). Moreover, long-term thermal niche conservatism during major climatic shifts has been demonstrated for a number of other taxa (e.g., Antell et al. 2021; Saupe et al. 2014). Therefore, at least for this case study, we consider the assumption safe.

There are significant differences between MTO and MTC, which prevent their direct comparison. The MTO captures the preserved part of an entire fish assemblage (Fig. 1), whereas MTC derives exclusively from the exploited component of fish assemblages. Therefore, MTO is affected by mortality and taphonomic processes, whereas MTC is influenced by fisheries targets and practices. Secondly, the catch composition reflects the snapshot of the exploited part of a modern assemblage that lives at the time of capture in the fishing ground or survey area, and MTC is calculated by summing up the catches over a relatively short period of time (e.g., yearly). On the other hand, death and fossil assemblages are time-averaged over much longer periods of time, usually exceeding the life span of the included fish species. Therefore, they represent accumulations of remains of individuals that died naturally typically over a period of decades to millennia (Albano et al. 2020; Nawrot et al. 2022). In addition, the sampling methodologies differ between living and death/fossil assemblages, making them incomparable at first instance. For example, a common method of fishing/surveying living fish assemblages is by net trawling, whereby the size of the captured fishes depends on the shape and dimensions of the net. Death/fossil assemblages are obtained by wet-sieving sediment samples of a given volume that corresponds to a limited surface area of the seabed. The mesh size of the sieve is usually 1 mm or smaller, so even the smallest species and otoliths of juvenile specimens are caught, but large fish individuals tend to be rare in death and fossil otolith assemblages. Finally, the MTC and MTO represent very different spatial scales, as the MTC is typically based on catch data encompassing large marine ecosystems or entire marine basins, while death/fossil otolith assemblages capture the composition of a local fish community. Thus, both the temporal and the spatial resolutions of the catch data and otolith assemblages differ by orders of magnitude. Therefore, MTO and MTC values should not be directly compared, but may be used independently to derive spatial and temporal trends, which are comparable.

The mean temperatures calculated from otolith death and fossil assemblages from the Eastern Mediterranean and northern Adriatic coasts reflect relatively well the regional differences in climate that would be expected to impact fish faunal composition. Thus, MTO represents a promising new proxy for tracking shifts in climatic affinity of fossil fish assemblages over large spatial and temporal scales. We highlight, however, that certain conditions must be met in such analyses: (a) sampling design should target specific questions (such as detecting fish stock changes at specific depths and through time in response to climate); (b) sample processing should be standardized; (c) a precise chronological framework is necessary to derive the most information out of such studies.

## Data Availability

All the data related to this paper are presented in the tables.
